# Revisiting Urological Injury Risks in Minimally Invasive Hysterectomy for Benign Indications: A Rare-Events Network Meta-Analysis with Sensitivity Analyses

**DOI:** 10.1007/s00192-026-06566-y

**Published:** 2026-03-25

**Authors:** Chiara Cassani, Arsenio Spinillo, Mattia Dominoni, Barbara Gardella

**Affiliations:** 1 Department of Clinical, Surgical, Diagnostic and Pediatric Sciences, University of Pavia, Piazzale Golgi 19, 27100 Pavia, Italy; 2 Unit of Obstetrics and Gynecology, IRCCS S. Matteo Foundation, Pavia, Italy

**Keywords:** Bladder injuries, Hysterectomy, Intraoperative complications, Laparoscopic hysterectomy, Network meta-analysis, Ureteral injuries

## Abstract

**Introduction and Hypothesis:**

The surgical approach to hysterectomy influences the risk of urological injuries, but the optimal technique for minimizing this risk remains unclear. Our objective was to evaluate, through a network meta-analysis (NMA) of randomized controlled trials (RCTs), the risk of intraoperative bladder and ureteral injuries among different hysterectomy techniques.

**Methods:**

We systematically searched Cochrane Central Register of Controlled Trials, MEDLINE, Embase, Google Scholar, ClinicalTrials.gov, World Health Organization International Clinical Trials Registry Platform, and European Clinical Trials Register (inception to 15 January 2025) for RCTs. NMA and pairwise meta-analyses were performed using penalized logistic regression and Bayesian models, accounting for rare events.

**Results:**

Out of 170 screened records, 63 RCTs involving 7470 women were included. Sixty-nine bladder injuries (9.2 out of 1000), 23 ureteral injuries (3.1 out of 1000), and 96 combined injuries (12.9 out of 1000) were reported. Compared with abdominal hysterectomy (AH), total laparoscopic hysterectomy (LH) showed a significantly higher risk of ureteral injury (odds ratio [OR] 7.74; 95% confidence interval [CI] 2.46–24.37), as did LH/laparoscopically assisted vaginal hysterectomy (LAVH; OR 7.73; 95% CI 2.42–24.7). LH and LH/LAVH also carried a higher risk of any urological injury than AH (OR 2.66 and 2.34 respectively). Estimated injury rates per 1000 were 9.5 (ureteral) and 18.1 (combined) for LH, versus 0.5 and 7.5 for AH. Meta-regression showed that the elevated risk with LH persisted over time.

**Conclusions:**

Laparoscopic hysterectomy and LH/LAVH are associated with a higher risk of ureteral/bladder injuries than AH. This increased risk has not declined over time, suggesting that the standardization and broader adoption of LH have not mitigated urological complications.

**Supplementary Information:**

The online version contains supplementary material available at https://doi.org/10.1007/s00192-026-06566-y

## Introduction

Currently, at least seven types or subtypes of hysterectomy performed for benign indications have been comparatively evaluated in randomized studies regarding complications, procedure duration, cost, hospital stay, and patient satisfaction [1–3]. These include abdominal hysterectomy (AH), laparoscopically assisted vaginal hysterectomy (LAVH), total laparoscopic hysterectomy (LH), vaginal hysterectomy (VH), single-port laparoscopic hysterectomy (SPLH), robot-assisted hysterectomy (RH), and vaginal natural orifice transluminal endoscopic surgery (vNOTES) [1–4]. Although single-port RH is currently performed, no concluded randomized trials compare this procedure with other approaches. Benign indications for hysterectomy typically include abnormal uterine bleeding, uterine fibroids, adenomyosis, endometriosis, chronic pelvic pain, pelvic organ prolapse, and pre-cancerous conditions [5]. In recent decades, the number of hysterectomies performed using laparoscopic, robot-assisted, and minimally invasive approaches has increased significantly [5, 6]. The primary advantages of these less invasive approaches include smaller incisions, reduced pain, faster recovery, and a lower risk of surgical-site infection [1–5]. However, well-known surgical complications of hysterectomy include intraoperative visceral injuries, such as bladder and/or ureteral damage [7]. The rates of intraoperative urological injuries requiring repair during hysterectomies for benign indications are low, typically between 0.5% and 1.5% of cases [1, 7]. However, given the large number of hysterectomies performed annually, even these low rates represent a significant number of patients. Bladder and ureteral injuries are linked to substantial morbidity and, in some cases, mortality; complications such as infection, fistula formation, renal impairment, ureteric stenting, and reimplantation surgeries contribute to a significant burden on patients’ quality of life and health care costs [7]. Given the rarity of these complications, most RCTs comparing the risk of complications between different types of hysterectomy are underpowered, and the results of such comparisons remain uncertain [1–3]. To address these issues, several pairwise meta-analyses pooling the results of different RCTs and comparing various hysterectomy approaches in terms of urological complications have been published in recent years [1–3]. However, performing a comparative meta-analysis of rare events is challenging because many included studies have single or double zero frequencies of the events [8]. For example, in a Cochrane review comparing ureteral injuries between LH and AH, all but one of the 10 studies included had at least one or both arms with zero frequencies [1]. Consequently, although the risk of ureteral injury appears higher in LH than in AH, the certainty of this finding is low. These challenges are common in both pairwise and network meta-analysis (NMA), the latter being an extension of classical meta-analysis that allows simultaneous multiple comparisons of different treatments [9]. The most common method of addressing the difficulty in the analysis of rare events is to add a 0.5 frequency to studies with a single arm with zero frequencies (a continuity correction) and to exclude studies with double-zero frequencies from the analysis [10]. However, this approach has been criticized because the addition of a 0.5 constant is arbitrary and can introduce bias, whereas the exclusion of studies with double-zero frequencies can bias the treatment effect and result in a loss of information by reducing sample size [8, 11, 12]. In recent years, several methods have been introduced to evaluate RCTs with rare events in both pairwise and NMA [13]. These procedures include Bayesian approaches with weakly informative priors, Bayesian binomial-normal models, and penalized logistic regression [14, 15]. These methods avoid both continuity corrections and the exclusion of double-zero studies. The objective of this NMA was to comparatively evaluate the risk of urological injuries associated with seven different types of hysterectomy using methods tailored to measure the risk of rare events in studies with a high rate of single- or double-zero frequencies.

## Materials and Methods

The study protocol was registered in the Prospective Register of Systematic Reviews (PROSPERO; registration number: CRD 42025635288). Data collection and analysis were conducted in accordance with the Preferred Reporting Items for Systematic Reviews and Meta-Analyses for Network Meta-Analyses (PRISMA-NMA) guidelines [16]. Using the Population, Intervention, Comparison, Outcomes, Study Design (PICOS) framework, the study population consisted of patients who had undergone various types of hysterectomy for benign indications in RCTs. The interventions compared included seven types of hysterectomies (AH, VH, LAVH, LH, RH, SPLH, and vNOTES). The primary outcomes evaluated were intraoperative bladder and/or ureteral injuries requiring surgical repair. RCTs were included only if the detection of visceral injuries was listed as a primary or secondary objective, or if the presence or absence of urological or visceral injuries was reported in the Results section.

The search strategy encompassed the following databases: Cochrane Central Register of Controlled Trials (CENTRAL), MEDLINE (Ovid), Embase (Embase.com), Google Scholar, the US National Institutes of Health Ongoing Trials Register (ClincalTrials.gov), the World Health Organization International Clinical Trials Registry Platform (apps.who.int/trialsearch/), and the European Clinical Trials Register (clinicaltrialsregister.eu).

**Fig. 1:**
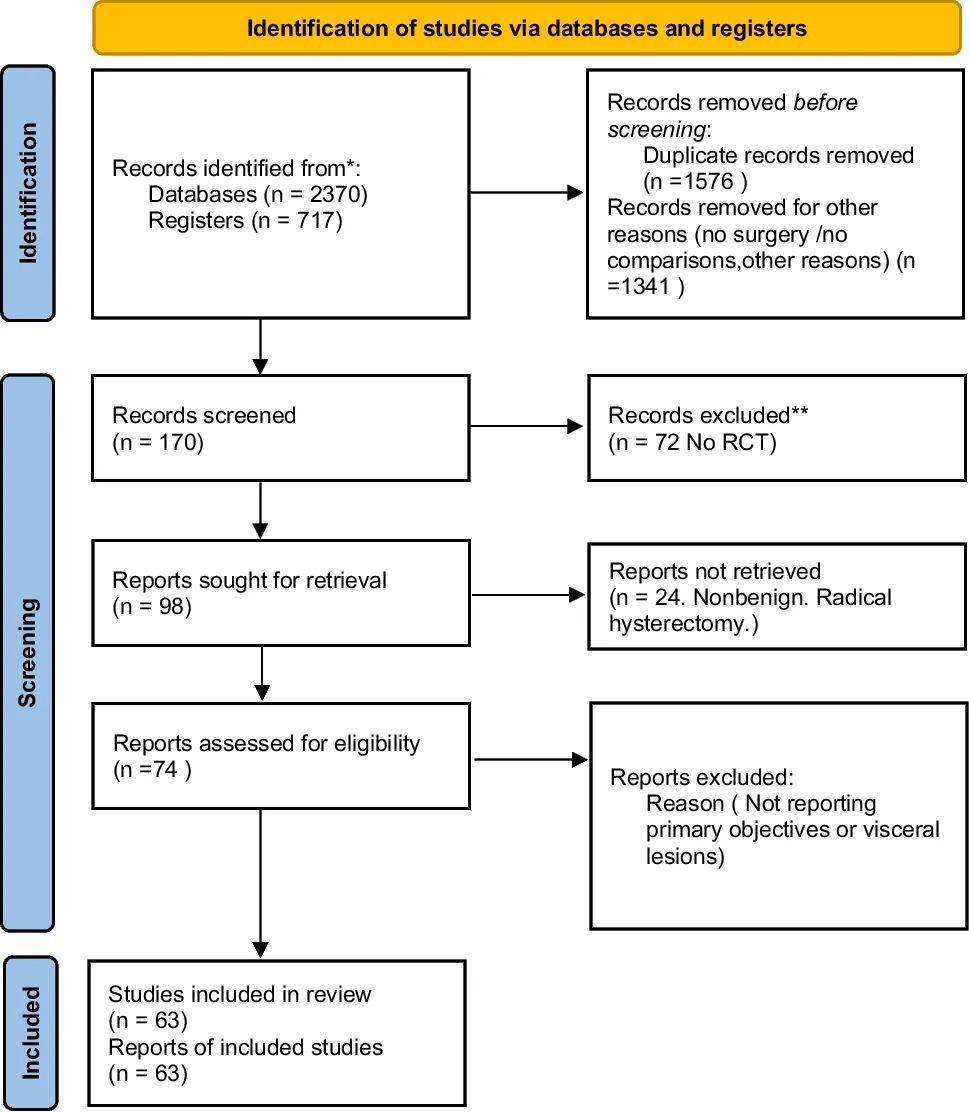
Preferred Reporting Items for Systematic Reviews and Meta-Analyses flow diagram of the studies included

Additionally, previously published traditional meta-analyses on this topic were reviewed. The search terms included "HYSTERECTOMY" combined with "RANDOMIZED" or "COMPLICATIONS." The PRISMA flow diagram of study selection and the checklist are provided in Fig. 1. Three reviewers (AS, MD, CC) independently screened the abstracts of the selected studies and extracted data from full-text articles. The extracted data were tabulated and compared for consistency. The final NMA included 63 randomized studies (Electronic Supplementary Material 1). The risk of bias (ROB) for individual studies was assessed using RoB 2 [17] by three reviewers (MD, CC, BG) under the supervision of a senior author (AS). Pooled rates of complications associated with each surgical approach were evaluated using the generalized linear mixed model in R (Meta subroutine). NMA and pairwise meta-analysis of rare events were conducted using the method proposed by Evrenoglou et al. [15], implemented in R (Netmeta subroutine). This method calculates ORs for rare events using a penalized logistic regression approach, incorporating a multiplicative overdispersion factor to account for heterogeneity in the final risk computation. This model performs better than traditional frequentist or Bayesian approaches, particularly when the proportion of studies with single- or double-zero event arms exceeds 25–30% [15]. The penalized logistic regression model avoids the need for continuity corrections or the exclusion of studies with zero counts in both arms. Consistency of the models was assessed by overall test and by comparing direct and indirect estimates for each comparison. Bayesian NMA models with narrow priors and half-normal distributions using the GEMCT program in R were used to confirm the results (sensitivity analysis) and to perform meta-regression [18]. In summary, pairwise meta-analysis with penalized logistic regression yielded reliable estimates for rare events, and these findings were confirmed by sensitivity analysis using a Bayesian binomial-normal hierarchical model. The year of publication of the included studies was included in meta-regression models as either a continuous or binary (studies published before 2010 vs 2010–2025) covariate to test whether the main results were influenced by the progressive adoption of newer hysterectomy techniques. In fact, approximately half of the studies were published before 2011, whereas the remaining half were published between 2011 and 2025. Following Owen et al. [19], the deviance information criterion (DIC) of models with and without the covariate was compared. A DIC difference greater than 3 suggests a significant effect of the covariate on the studied outcome. Finally, direct pairwise meta-analysis results obtained by penalized logistic regression were confirmed using a Bayesian binomial-normal hierarchical model as described by Günhan et al. [14] (sensitivity analysis; subroutine Metastan in R). Before starting the statistical analysis, a preliminary sample size was computed with reference to bladder/ureteral injuries in a pairwise comparison. Assuming that bladder/ureteral injuries occur in about 1–1.5% of hysterectomies [1, 7], a sample size of approximately 2500 participants for each comparison was needed to detect an OR of 2–2.5 in a random-effects model with low heterogeneity (0.2) at the usual type I error of 0.05 and type II error of 0.2 (Subroutine RTSA, R).

## Results

**Fig. 2:**
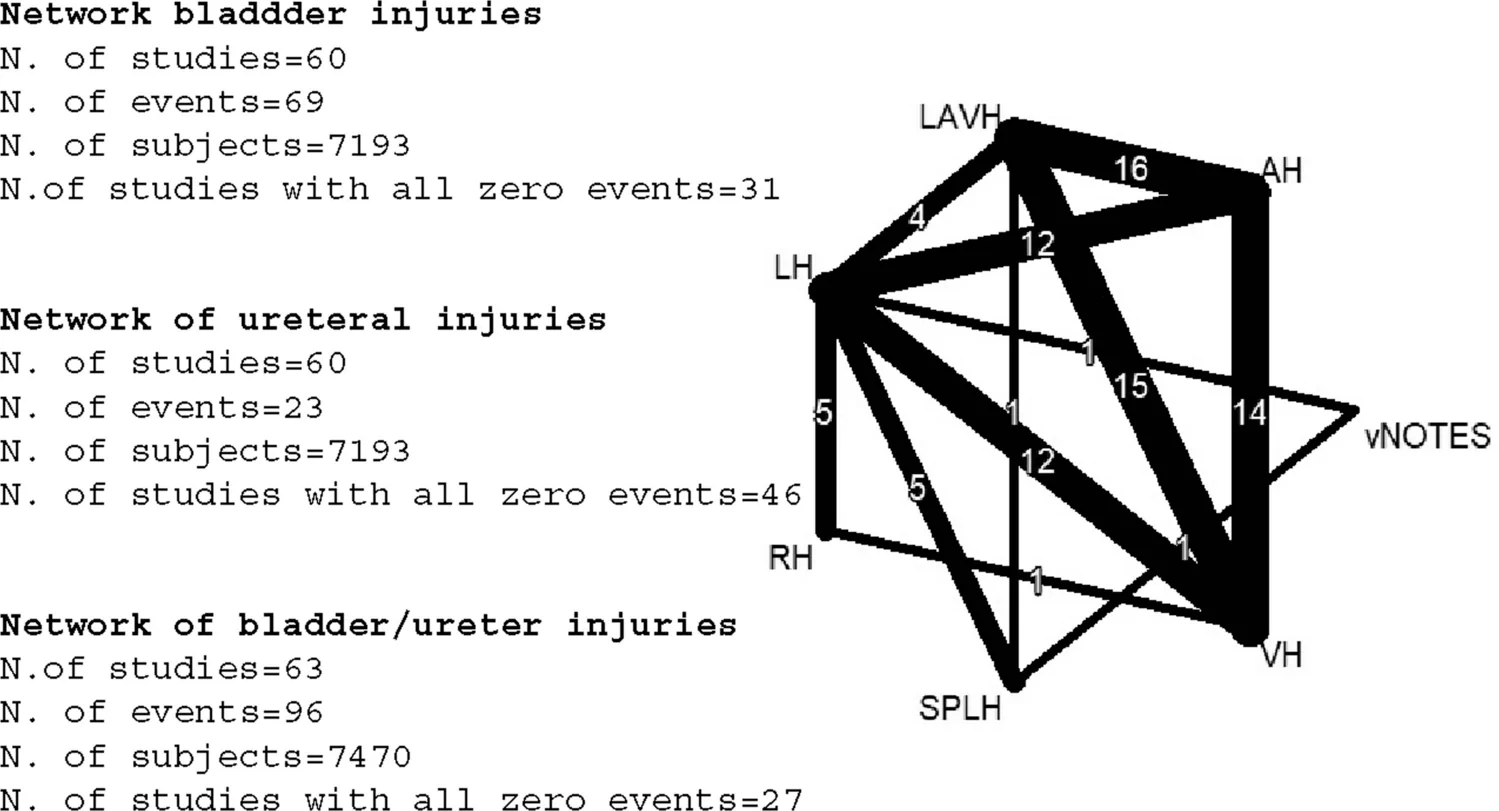
Network of the studies included. *AH* abdominal hysterectomy, *LAVH* laparoscopically assisted vaginal hysterectomy, *LH* laparoscopic hysterectomy, *RH* robot-assisted hysterectomy, *SPLH* single-port laparoscopic hysterectomy, *VH* vaginal hysterectomy, *vNOTES* vaginal natural orifice transluminal endoscopic surgery

Out of the 63 studies included in the analysis, 33 (52.4%) were published before 2010, and 30 (47.6%) were published in or after 2010 (Electronic Supplementary Material 1). The network included seven types of interventions (AH, LAVH, LH, VH, RH, SPLH, vNOTES), with 51 studies involving two-arm comparisons, 12 involving three-arm comparisons, and a total of 7470 women (Fig. 2). In 3 studies (*n* = 277, events = 4; Electronic Supplementary Material 1) the outcome of interest was generically reported as urological damage. These cases were counted only in the group of bladder/ureteral injuries. The overall number of dropouts reported in the primary studies was low (*n* = 110) and these subjects were already excluded from the results of the trials included.

### Bladder Injuries

**Fig. 3:**
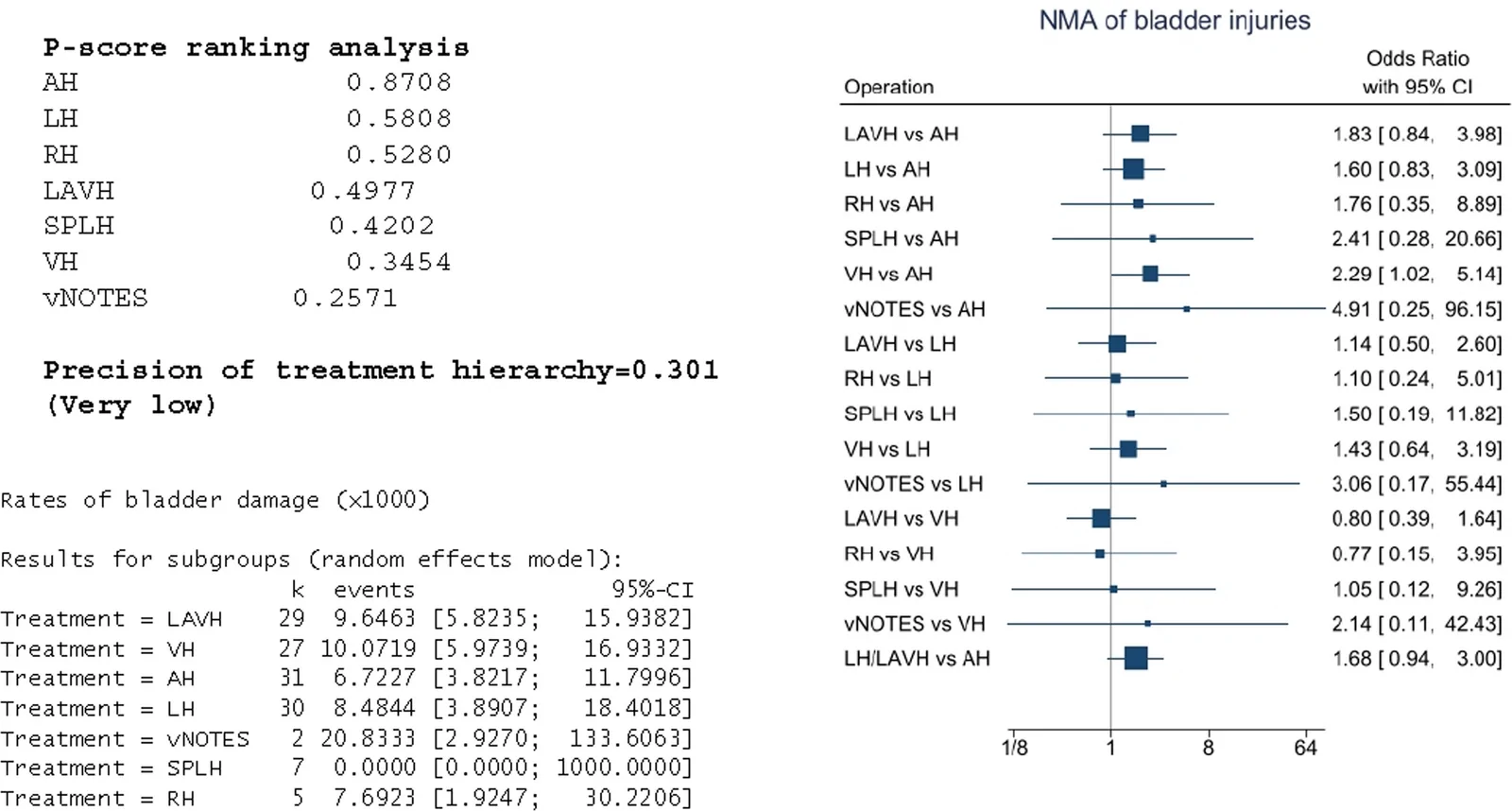
Network of the studies included, estimated prevalence of bladder injuries, and results of network meta-analysis. *AH* abdominal hysterectomy, *CI* confidence interval, *LAVH* laparoscopically assisted vaginal hysterectomy, *LH* laparoscopic hysterectomy, *NMA* network meta-analysis, *RH* robot-assisted hysterectomy, *SPLH* single-port laparoscopic hysterectomy, *VH* vaginal hysterectomy, *vNOTES* vaginal natural orifice transluminal endoscopic surgery

The estimates of bladder injury per 1000 stratified by the type of operation are reported in Fig. 3 There were a total of 69 bladder injuries among the 7193 subjects (9.6 per 1000), and no events were reported in 27 out of the 60 studies. The crude rates of bladder injury per 1000 for LAVH, VH, AH, LH, vNOTES, and RH were 15 out of 1555 (9.6), 14 out of 1390 (10.1), 12 out of 1785 (6.7), 25 out of 1867 (13.3), 1 out of 48 (20.8), and 2 out of 260 (7.6) respectively. No cases of bladder injuries were reported among the 288 cases of SPLH across seven studies. In the network analysis, the risk of bladder injury was slightly higher in VH than in AH, but this result was not confirmed in pairwise meta-analysis using either penalized logistic (OR 2.19, 95% CI 0.71–2.63) or Bayesian binomial-normal models (OR 2.43, 95% CI 0.54–14.9; Electronic Supplementary Material 2). No significant differences in the outcome were observed between the other surgical approaches. Both global test (*p* = 0.9) and the comparison of direct and indirect evidence were not significant, suggesting model consistency (Electronic Supplementary Material 2), and most of the evidence came from direct comparisons. Ranking analysis of data suggests that AH is the method with the lowest likelihood of bladder injury but the precision of hierarchy was very low (Fig. 3). The inclusion of the year of publication as a continuous covariate in meta-regression had no significant effect on the DIC (105.8 without and 106.6 with the covariate), indicating a nonsignificant effect of the publication year on the model.

### Ureteral Injuries

**Fig. 4:**
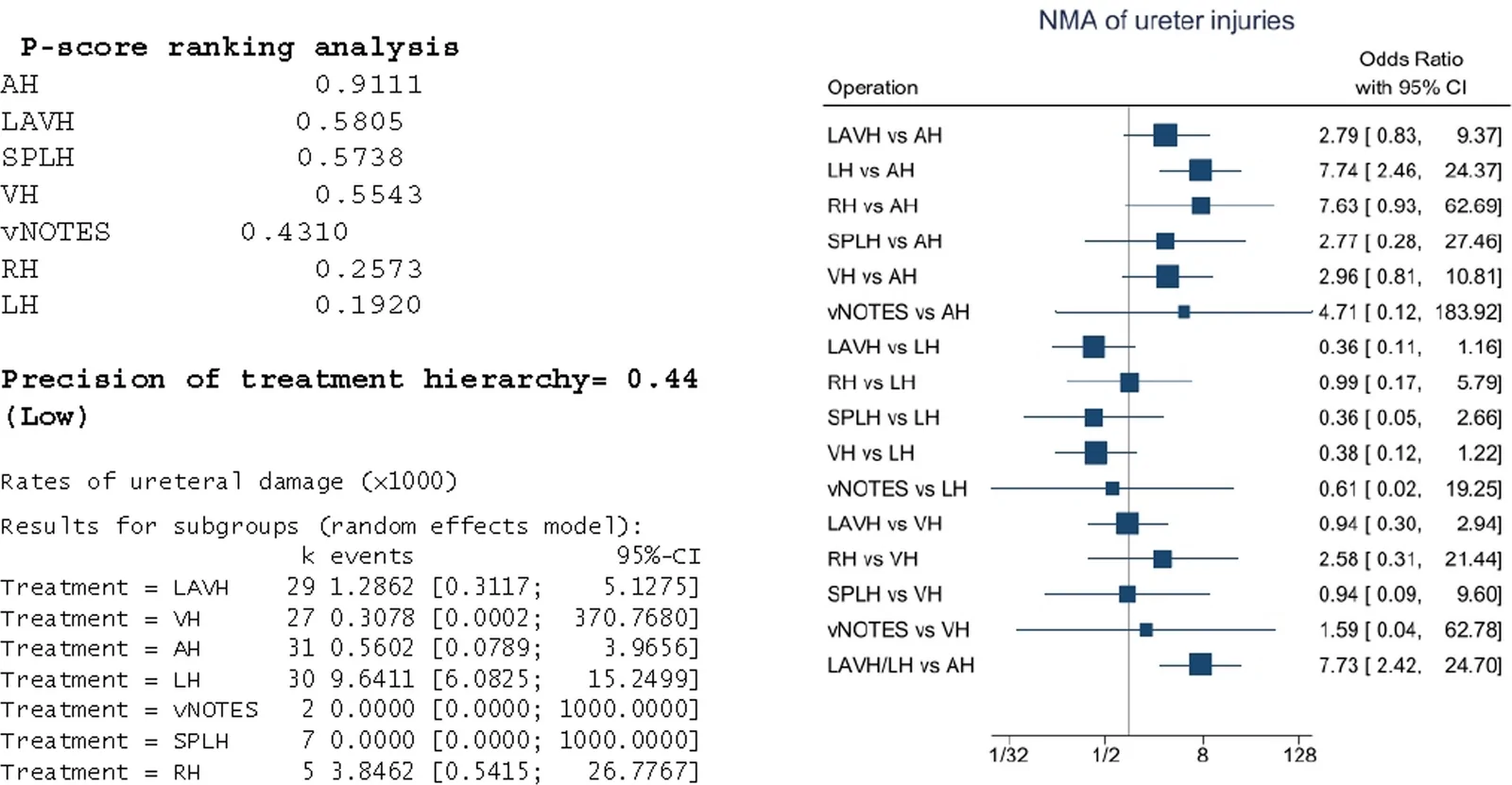
Network of the studies included, estimated prevalence of ureter injuries, and results of network meta-analysis. *AH* abdominal hysterectomy, *CI* confidence interval, *LAVH* laparoscopically assisted vaginal hysterectomy, *LH* laparoscopic hysterectomy, *NMA* network meta-analysis, *RH* robot-assisted hysterectomy, *SPLH* single-port laparoscopic hysterectomy, *VH* vaginal hysterectomy, *vNOTES* vaginal natural orifice transluminal endoscopic surgery

The network of ureteral injuries is reported in Fig. 4 and includes a total of 23 injuries among the 7193 subjects (3.2 per 1000). There were 46 out of 60 studies with no events. Out of the 23 cases of ureteral injuries, 18 were reported in LH and 2 in LAVH operations. In the NMA, LH and LAVH/LH hysterectomies were associated with an OR of ureteral injury more than seven times higher than that of abdominal hysterectomies. This increased risk of ureteral injuries in LAVH/LH compared with AH was confirmed in pairwise meta-analysis using both penalized logistic (OR 7.58, 95% CI 2.29–25.16) and Bayesian binomial-normal models (OR 6.3, 95% CI 1.65–35.2; Electronic Supplementary Material 3). Node-split analysis was not significant, suggesting that the model was consistent and most of the information derived from direct comparisons (Electronic Supplementary Material 3). Compared with AH, the number needed to harm (NNTH) of LH operations in terms of ureteral injuries was 267 (95% CI 79–1192). Ranking analysis confirmed that AH and LH were the procedures carrying the lowest and highest risks of ureteral injury, although the precision of treatment hierarchy was low (Fig. 4). In meta-regression, the DIC values were 48.9 for the model without the year of publication and 49.1 for the model with the year included as a continuous covariate, indicating no significant impact. Although the CIs were wide, the ORs of ureteral injuries in LAVH/LH compared with AH were 10.6 (95% CI = 1.9–126) for studies published before 2010 and 12 (95% CI = 2–194) for studies published between 2010 and 2024.

### Bladder/Ureteral Injuries

**Fig. 5:**
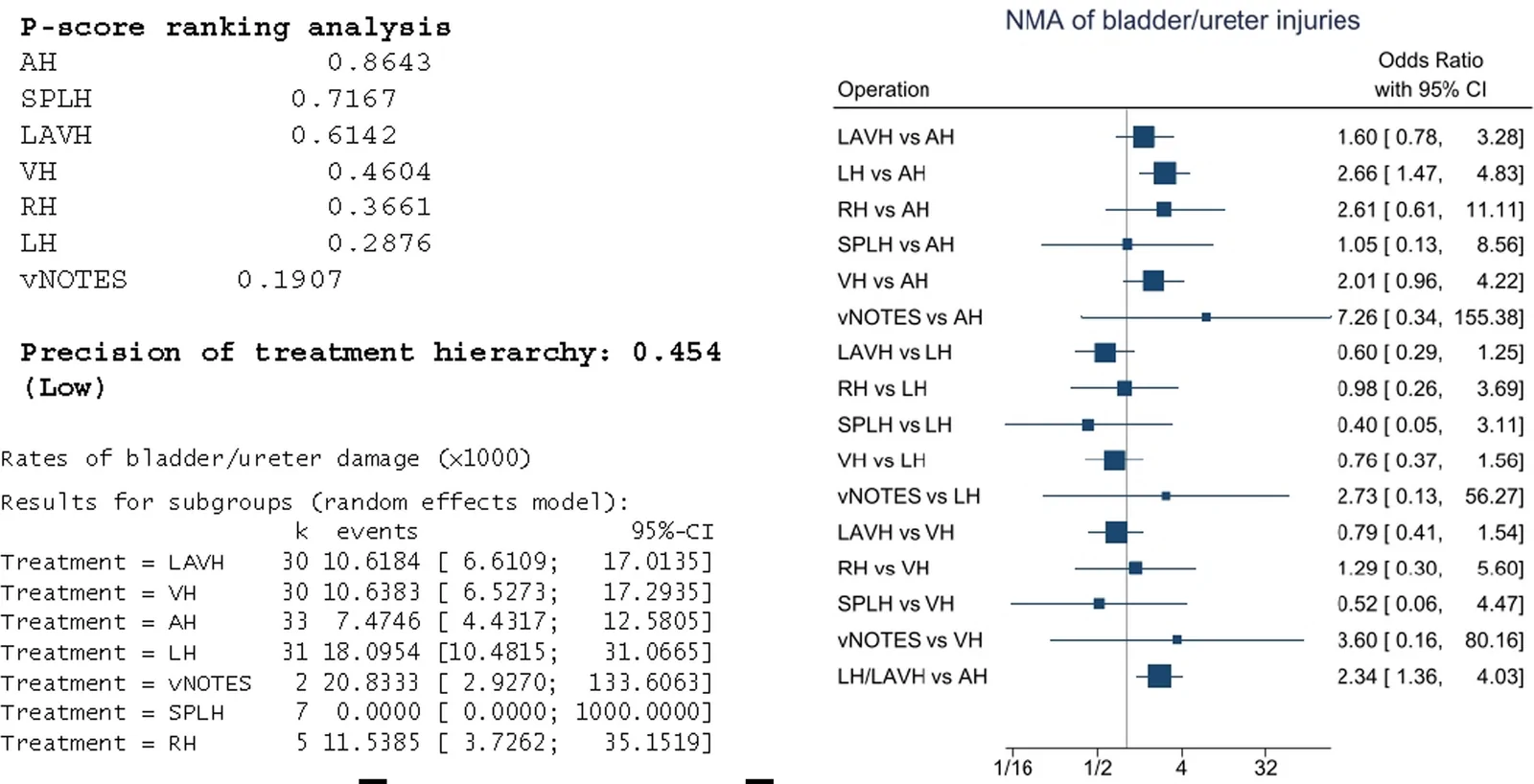
Network of the studies included, estimated prevalence of bladder/ureter injuries, and results of network meta-analysis. *AH* abdominal hysterectomy, *CI* confidence interval, *LAVH* laparoscopically assisted vaginal hysterectomy, *LH* laparoscopic hysterectomy, *NMA* network meta-analysis, *RH* robot-assisted hysterectomy, *SPLH* single-port laparoscopic hysterectomy, *VH* vaginal hysterectomy, *vNOTES* vaginal natural orifice transluminal endoscopic surgery

Bladder and/or ureteral injuries were reported in 96 out of the 7470 patients in the network (12.9 per 1000), and the estimated prevalence, stratified by the type of hysterectomy, is reported in Fig. 5. There were 27 studies with no events. The ORs of bladder/ureteral injuries were significantly higher in LH (OR 2.66, 95% CI 1.47–4.83) and LAVH/LH (OR 2.34, 95% CI 1.36–4.03) than in AH, both in NMA and direct pairwise meta-analyses. Specifically, the ORs of bladder/ureteral injuries in LAVH/LH compared with AH in pairwise meta-analyses were 2.31 (95% CI 1.32–4.05) and 2.32 (95% CI 1.2–4.7) in penalized logistic and Bayesian binomial-normal models respectively (Electronic Supplementary Material 4). The overall sample size of this pairwise comparison included 3257 subjects. DIC values for the model with and without the year of publication as a continuous covariate were 125 and 123.3 respectively. The ORs of bladder/ureteral injuries in LAVH/LH compared with AH were 2.5 (95% CI 1.11–6.4) for studies published before 2010 and 2.6 (95% CI 1.14–6.2) for studies published between 2010 and 2024. As with the other comparisons, most of the information on the effect size was derived from direct comparisons, and global test (*p* = 0.98) and node-split analysis suggested consistency of the model (Electronic Supplementary Material 3). The NNTH of LH operations compared with AH was 82 (95% CI 36–286). Finally, ranking analysis confirmed that AH was the method with the lowest probability of bladder/ureteral injury, but, even for overall analysis, the precision of hierarchy was low.

### Risk of Bias Analysis

The results of ROB assignment are reported in Electronic Supplementary Materials 5 and 6. Of the 63 studies included, 8 (12.7%) were judged to be at a high ROB, 36 (57.1%) at an intermediate ROB, and 27 (42.9%) at a low ROB. Deviation from intended interventions was the most common (25 out of 63 studies) bias recorded. After exclusion from the analysis of the 8 studies at a high ROB, the ORs of ureter and bladder/ureter injuries in LH/LAVH compared with AH were 5.9 (95% CI 1.8–19.2) and 2.5 (95% CI 1.35–4.49) respectively.

## Discussion

The results of the present study confirm that, in RCTs of intraoperative complications of hysterectomies for benign indications, LH and LAVH operations are associated with an increased risk of ureteral and bladder/ureteral injuries compared with classic AH. This increased risk was confirmed through both network and pairwise analyses, utilizing different methods specifically aimed at evaluating the risk of rare events. In meta-regression, the increased likelihood of urological injuries was independent of the year of publication, suggesting that this risk might remain consistent during the progressive diffusion and standardization of LH. No additional differences in the outcomes studied were found between other types of hysterectomy and AH or between all other types of surgical approaches.

The findings of this study have important clinical implications. Although LH and LAVH provide advantages such as faster recovery, reduced blood loss, and shorter hospitalization, they remain associated with a higher risk of bladder and ureteral injuries than AH. A crude rate of bladder/ureter injuries according to surgical approach is reported in Electronic Supplementary Material 7: based on current estimates, the widespread use of laparoscopic approaches may result in one more urological damage every 82 procedure. Even if infrequent, these complications can result in significant morbidity, reoperation, and long-term sequelae. Consequently, patients should be counseled transparently about both the benefits and risks of minimally invasive techniques to support truly informed decision making.

### Comparison with Existing Literature

Several pairwise meta-analyses on the impact of different types of hysterectomy for benign indications on intraoperative complications, duration of procedure, blood loss, hospital stay, satisfaction, quality of life, and major long-term complications have been published in recent years [1–4, 20, 21]. The main conclusions of these studies were that, when feasible, VH should be preferred over AH owing to fewer complications and a faster return to normal activities. Other recommendations suggested that LH should be preferred over AH because of shorter hospital stays, lower infection rates, and better cosmetic outcomes [1–4]. However, these advantages should be weighed against an increased risk of urological injuries, which, given their rarity, were associated with low certainty [1]. The increased risk of urinary tract damage associated with LH as compared with AH was based on the analysis of 10 studies (9 with one- or double-zero frequencies of the event) for ureter injuries and 17 studies (13 with one- or double-zero frequencies of the event) for bladder/ureter injuries [1]. The results of the present study, conducted using appropriate methods to assess the risk of rare events, confirm the increased risk of ureteral and bladder/ureteral injuries associated with LH compared with AH. This increased risk has remained stable over the last few decades, suggesting that the progressive diffusion and standardization of LH have not reduced the risk of associated urological injuries. Data comparing LH or AH with more recent approaches (e.g., RH, vNOTES, SPLH) are too limited to draw definitive conclusions. To the best of our knowledge, only one network meta-analysis on the effect of different types of hysterectomy on short-term patient outcomes has been published [21]. This analysis included a large number of Chinese RCTs, utilized standard methods of NMA, excluded AH operations, and evaluated visceral injuries collectively without differentiating the organs involved. The study concluded that VH was associated with a higher rate of visceral injuries than LAVH, although this effect was evident only in Chinese RCTs.

### Strengths and Limitations

The main strengths of this manuscript include the use of different methods specifically introduced in the literature to evaluate the risk of rare events and their application to both pairwise and NMA. Standard published meta-analyses of the risk of visceral injuries associated with different types of hysterectomy have typically used conventional methods, such as the Peto approach, which involve the application of a correction factor and the exclusion of studies with a zero frequency of the event in cases and controls. This is particularly relevant for ureteral injuries, which occur in less than 1% of cases [1–4]. Additional strengths include the inclusion of studies with unambiguous reporting of visceral injuries, either in the objectives or results of the trial. Finally, the use of meta-regression in Bayesian models allowed us to assess whether the progressive diffusion of the laparoscopic approach had a significant impact on the risk of visceral injuries.

The main limitations of this study include the low number of trials and subjects involving relatively new hysterectomy approaches (e.g., SPLH, vNOTES, and RH), which precluded definitive conclusions about the associated risk of urological injuries compared with LH or the older AH approach. Additionally, although only randomized studies were included, it was impossible to stratify or adjust for surgical indications or other factors such as uterine size, patient characteristics (e.g., age, obesity, or associated diseases), surgeon experience or hospital volume. Moreover, all included studies assessed only intraoperative urological complications or early postoperative events that were clinically evident without the routine use of cystoscopy or other urological procedures. Therefore, the possibility that some bladder and/or ureteral injuries went undetected cannot be excluded. Finally, all RCTs focused exclusively on early complications, with no data reported on delayed urological complications or morbidity related to their management. All these factors are likely to influence the overall rates of urological complications [1, 2, 5].

### Implications and Conclusions

Compared with AH, laparoscopic and laparoscopically assisted procedures increase the risk of bladder and ureteral injuries, highlighting the need to balance minimally invasive benefits with the potential for rare but clinically significant complications.

## Supplementary Information

Below are the links to the electronic supplementary material.

Supplementary file1 (DOCX 45 KB)

Supplementary file2 (PPTX 312 KB)

Supplementary file3 (PPTX 329 KB)

Supplementary file4 (PPTX 331 KB)

Supplementary file5 (PDF 242 KB)

Supplementary file6 (PDF 400 KB)

Supplementary file7 (DOCX 15 KB)

## Data Availability

The datasets generated and analysed during the current study are available from the corresponding author on reasonable request.

## Declarations

### Registration

PROSPERO (registration number: CRD42025635288, 21 January 2025).
