# Supplementary material for: Revisiting Urological Injury Risks in Minimally Invasive Hysterectomy for Benign Indications: A Rare-Events Network Meta-Analysis with Sensitivity Analyses
Source: Int Urogynecol J. 2026 Mar 25;37(9):2791–8. doi: 10.1007/s00192-026-06566-y (PMC13649814; doi:10.1007/s00192-026-06566-y)
Supplement: Supplementary file 1 [file 192_2026_6566_MOESM5_ESM.pdf]

Intention-to-treat	Unique ID	Study ID	Experimental	Comparator	Outcome	Weight	D1	D2	D3	D4	D5	Overall
	V1	Agostini et al, 2006	NA	NA	NA	1	+	+	+	!	!	!
	V2	Allam et al, 2015	NA	NA	NA	1	+	+	+	+	+	+
	V3	Baekelandt et al, 20	NA	NA	NA	1	+	+	+	+	+	+
	V4	Balakrishnan et al, :	NA	NA	NA	1	+	+	+	+	+	+
	V5	benassi et al, 2002	NA	NA	NA	1	+	!	+	+	+	!
	V6	Candiani et al, 2009	NA	NA	NA	1	+	+	+	!	+	!
	V7	Chakraborty et al, :	NA	NA	NA	1	!	!	+	+	+	!
	V8	Chen et al, 2011	NA	NA	NA	1	+	!	+	+	+	!
	V9	Chrysostomou et al	NA	NA	NA	1	-	-	+	+	+	-
	V10	Chung et al, 2015	NA	NA	NA	1	+	+	+	+	+	+
	V11	Darai et al, 2001	NA	NA	NA	1	+	!	+	+	+	!
	V12	Deimling et al, 2016	NA	NA	NA	1	+	+	+	+	+	+
	V13	Drahonovsky et al,	NA	NA	NA	1	+	+	+	+	+	+
	V14	Eggemann et al, 20	NA	NA	NA	1	+	+	+	!	+	!
	V15	Ekanayake et al, 20	NA	NA	NA	1	+	-	+	+	+	-
	V16	Falcone et al, 1999	NA	NA	NA	1	+	+	+	!	+	!
	V17	Ferrari et al, 1999	NA	NA	NA	1	+	+	+	!	+	!
	V18	Garry et al, 2004	NA	NA	NA	1	+	!	+	+	+	!
	V19	Garry et al, 2004	NA	NA	NA	1	+	!	+	+	+	!
	V29	Ghezzi et al, 2010	NA	NA	NA	1	+	!	+	+	+	+
	V30	Hendawy et al, 201	NA	NA	NA	1	+	!	+	!	+	!
	V20	Hwang et al, 2002	NA	NA	NA	1	+	+	+	+	+	+
	V21	Jung et al, 2011	NA	NA	NA	1	+	!	+	+	+	!
	V22	Kim et al, 2015	NA	NA	NA	1	+	+	+	+	+	+
	V23	Kluivers et al, 2006	NA	NA	NA	1	+	!	+	+	+	!
	V24	Kongwattanakul et	NA	NA	NA	1	+	+	+	+	+	+
	V25	Langebrekke et al, 1	NA	NA	NA	1	+	!	+	+	+	!
	V27	Long et al, 2002	NA	NA	NA	1	!	!	+	+	+	!
	V28	Lönnerfors et al, 20	NA	NA	NA	1	+	+	+	+	+	+
	V31	Mara et al, 1999	NA	NA	NA	1	+	+	+	+	+	+
	V32	Martinez-martinez	NA	NA	NA	1	-	!	+	+	+	-
	V33	Miskry et al, 2003	NA	NA	NA	1	+	+	+	+	+	+
	V34	Mohammed et al, :	NA	NA	NA	1	+	-	+	+	+	-
	V35	Muzil et al, 2007	NA	NA	NA	1	+	+	+	+	+	+
	V36	Nanavati et al, 2016	NA	NA	NA	1	!	-	+	+	+	-
	V37	Olsson et al, 1996	NA	NA	NA	1	+	+	+	+	+	+
	V38	ottosen et al, 2000	NA	NA	NA	1	+	+	+	+	+	+
	V39	panda et al, 2015	NA	NA	NA	1	!	-	+	!	+	-
	V40	Paraiso et al, 2013	NA	NA	NA	1	+	+	+	+	+	+
	V41	Park et al, 2021	NA	NA	NA	1	+	+	+	+	+	+
	V42	Parveen et al, 2013	NA	NA	NA	1	-	!	+	!	+	-
	V43	Perino et al, 1999	NA	NA	NA	1	!	+	+	+	!	!
	V44	Person et al, 2006	NA	NA	NA	1	+	+	+	+	!	!
	V45	Raju et al, 1994	NA	NA	NA	1	+	+	+	+	+	+
	V46	Ribeiro et al, 2003	NA	NA	NA	1	!	+	+	+	+	!
	V47	Richardson et al, 19	NA	NA	NA	1	+	+	+	+	+	+
	V48	Roy et al, 2011	NA	NA	NA	1	!	+	+	+	+	!
	V49	Roy et al, 2012	NA	NA	NA	1	!	+	+	+	!	!
	V50	Sarlos et al, 2012	NA	NA	NA	1	+	+	+	+	+	+
	V51	schutz et al, 2002	NA	NA	NA	1	+	+	+	+	+	+
	V52	Serracchioli et al, 20	NA	NA	NA	1	+	+	+	+	+	+
	V53	Sesti et al, 2008	NA	NA	NA	1	+	+	+	+	+	+
	V54	Sesti et al, 2008b	NA	NA	NA	1	+	+	+	+	+	+
	V55	Sesti et al, 2014	NA	NA	NA	1	+	+	+	+	+	+
	V56	Song et al, 2013	NA	NA	NA	1	+	+	+	+	+	+
	V57	Summit et al, 1992	NA	NA	NA	1	+	!	!	+	+	!
	V58	Summit et al, 1998	NA	NA	NA	1	+	+	+	+	!	!
	V59	Yuen et al, 1998	NA	NA	NA	1	+	!	+	+	+	!
	V60	Zhu et al, 2009	NA	NA	NA	1	!	!	+	+	!	!
	V61	Tsai et al, 2003	NA	NA	NA	1	+	!	+	+	+	!
	V62	Tormena et al, 2017	NA	NA	NA	1	!	!	+	+	+	!
	V63	Wakhloo et al, 2015	NA	NA	NA	1	-	!	+	!	+	-

+ Low risk
! Some concerns
- High risk

D1 Randomisation process
D2 Deviations from the intended interventions
D3 Missing outcome data
D4 Measurement of the outcome
D5 Selection of the reported result
