# Supplementary material for: Revisiting Urological Injury Risks in Minimally Invasive Hysterectomy for Benign Indications: A Rare-Events Network Meta-Analysis with Sensitivity Analyses
Source: Int Urogynecol J. 2026 Mar 25;37(9):2791–8. doi: 10.1007/s00192-026-06566-y (PMC13649814; doi:10.1007/s00192-026-06566-y)
Supplement: Supplementary file 3 [file 192_2026_6566_MOESM1_ESM.docx]

| **Author** | **Year** | **Country** | **Population: main indications** | **Intervention** | **Number of patients** | **Number of bladder injuries** | **Number of ureteral injuries** | **Number of bladder/ureteral injuries** |
| --- | --- | --- | --- | --- | --- | --- | --- | --- |
| Agostini | 2006 | France | Fibroma or Myoma, Adenomyosis; | LAVH  VH | 24  24 | 0  0 | 0  0 | 0  0 |
| Allam | 2015 | Egypt | Benign gynecological diseases, Uterus size ≤16 weeks; | VH  AH  LH | 30  30  30 | 0  1  0 | 0  0  0 | 0  1  0 |
| Baekelandt | 2018 | Belgian | Fibroma or Myoma; Adenomyosis; Hyperplasia; Dysfunctional uterine bleeding; BRCA-positive breast cancer | LH  vNOTES | 35  35 | 0  1 | 0  0 | 0  1 |
| Balakrishnan | 2016 | India | Symptomatic uterine fibroids, adenomyosis, high-grade cervical dysplasia, dysfunctional uterine bleeding, atypical endometrial hyperplasia, BRCA-positive women 45 years of age or older | AH  VH | 150  150 | 0  0 | 0  0 | 0  0 |
| Benassi | 2002 | Italy | Benign gynecological diseases; US estimated uterine volume ≥ 200 ml and < 1300 ml | VH  AH | 60  59 | 0  0 | 0  0 | 0  0 |
| Candiani | 2009 | Italy | Fibroma or Myoma; Adenomyosis; Hyperplasia; Ovarian cyst | LH  VH | 30  30 | 0  0 | 0  0 | 0  0 |
| Chakraborty | 2011 | India | Benign gynecological diseases | VH  AH | 100  100 | 1  0 | 0  0 | 1  0 |
| Chen | 2011 | China (Taiwan) | Fibroma or Myoma; Adenomyosis; Hyperplasia; CIN or cervical disease | LAVH  SPLH | 50  50 | 0  0 | 0  0 | 0  0 |
| Chrysostomou | 2021 | South Africa | Fibroma or Myoma; Adenomyosis; Hyperplasia; Dysfunctional uterine bleeding; uterus size <12 weeks | LAVH  VH | 76  151 | 1  3 | 0  0 | 1  3 |
| Chung | 2015 | Korea | Fibroma or Myoma; Adenomyosis; Hyperplasia; Ovarian cyst; CIN or cervical disease; endometriosis or endometrial disease | LH  SPLH | 29  29 | 0  0 | 0  0 | 0  0 |
| Daraï | 2001 | France | Fibroma or Myoma; Dysfunctional uterine bleeding; Uterine adnexal disease; Endometriosis or endometrial disease; Dysmenorrhea or Pelvic pain; uterine  size larger than 280 g | LAVH  VH | 40  40 | 1  0 | 0  0 | 1  0 |
| Deimling | 2017 | USA | Fibroma or Myoma; Dysfunctional uterine bleeding; Endometriosis or endometrial disease; Dysmenorrhea or Pelvic pain | LH  RH | 72  72 | 0  0 | 0  1 | 0  1 |
| Drahonovsky | 2010 | Czech Republic | Fibroma or Myoma; Hyperplasia; Dysfunctional uterine bleeding | VH  LAVH  LH | 40  44  41 | 1  1  1 | 0  0  1 | 1  1  2 |
| Eggemann | 2018 | Germany | Benign gynecological diseases | LAVH  VH | 95  97 | 1  1 | 0  0 | 1  1 |
| Ekanayake | 2021 | Sri Lanka | Benign gynecological diseases; uterus size ≤14 weeks | VH  AH  LH | 49  49  49 | 2  0  0 | 0  0  1 | 2  0  1 |
| Falcone | 1999 | USA | Benign gynecological diseases; maximal size 2 cm below the umbilicus. | AH  LAVH | 24  24 | 0  0 | 0  0 | 0  0 |
| Ferrari | 2000 | Italy | Benign gynecological diseases; US estimated uterine volume ≥ 500 ml and < 1500 ml | LAVH  AH | 31  31 | 0  0 | 0  0 | 0  0 |
| Garry | 2004 a | South Africa | Endometriosis or endometrial disease; Dysmenorrhea or Pelvic pain; Previous failure of ablation treatment; uterus size ≤12 weeks | LH  AH | 584  292 | 15  3 | 5  0 | 20  3 |
| Garry | 2004 b | UK/ South Africa | Fibroma or Myoma; Dysfunctional uterine bleeding; Endometriosis or endometrial disease; Dysmenorrhea or Pelvic pain; Previous failure of ablation treatment; uterus size ≤12 weeks | LAVH  VH | 336  168 | 3  2 | 1  0 | 4  2 |
| Ghezzi | 2010 | Italy | Fibroma or Myoma; Adenomyosis; Hyperplasia; CIN or cervical disease; Uterine adnexal disease; Uterus size ≤14 weeks | LH  VH | 41  41 | 0  0 | 0  0 | 0  0 |
| Hendawy | 2018 | Egypt | Benign gynecological diseases; large uteri size≥12weeks and <16weeks | VH  LAVH | 30  30 | 0  0 | 0  0 | 0  0 |
| Hwang | 2002 | China (Taiwan) | Uterine fibroids larger than 6 cm in diameter or uteri estimated to weigh at least 450g | VH  LH  AH | 30  30  30 | 0  0  0 | 0  0  0 | 0  0  0 |
| Jung | 2011 | Korea | Fibroma or Myoma; CIN or cervical disease; Uterine adnexal disease; Endometriosis or endometrial disease; Uterus size <12 weeks | LH  SPLH | 68  30 | 0  0 | 0  0 | 0  0 |
| Kim | 2015 | Korea | Fibroma or Myoma; Adenomyosis; Uterus size ≤18 weeks | LH  SPLH | 126  125 | 0  0 | 1  0 | 1  0 |
| Kluivers | 2007 | Netherlands | Benign gynecological diseases; large uteri size≥12weeks and <16weeks | LH  AH | 27  32 | 1  2 | 1  0 | 2  2 |
| Kongwattanakul | 2012 | Thailand | Benign gynecological diseases; uterus size ≤16 weeks | LAVH  AH | 25  25 | 1  0 | 0  0 | 1  0 |
| Langebrekke | 1996 | Norway | Benign gynecological diseases; uterus size ≤12 weeks | LH  AH | 46  54 | 1  1 | 2  0 | 3  1 |
| Long | 2002 | China (Taiwan) | Benign gynecological diseases; uterus weighs < 200 g | LH  LAVH | 41  60 | 1  2 | 2  1 | 3  3 |
| Lönnerfors | 2015 | Sweden | Benign gynecological diseases; uterus size ≤16 weeks | VH  LH  RH | 25  36  61 | 0  1  1 | 0  0  0 | 0  1  1 |
| Lumsden | 2000 | UK | Benign gynecological diseases; uterine size >14 weeks or a requirement for oophorectomy | LH  AH | 95  95 | 1  0 | 1  1 | 2  1 |
| Marana | 1999 | Italy | Benign gynecological diseases; uterine weight more than 280 g but less than 700 g | LAVH  AH | 58  58 | 1  0 | 0  0 | 1  0 |
| Martinez-Maestre | 2014 | Spain | Benign gynecological diseases, uterine length ≤16 cm | LH  RH | 54  51 | 1  1 | 0  0 | 1  1 |
| Miskry | 2003 | UK | Dysfunctional uterine bleeding, Fibroids, Pelvic pain | AH  VH | 18  18 | 0  0 | 0  0 | 0  0 |
| Mohammed | 2017 | Egypt | Benign gynecological diseases; large uterine weight more than 280 g | LAVH  VH | 25  25 | 1  1 | 0  0 | 1  1 |
| Muzii | 2007 | Italy | Benign gynecological diseases; uterine weight more than 280 g but less than 700 g | LAVH  AH | 40  41 | 0  0 | 0  0 | 0  0 |
| Nanavati | 2016 | India | Benign gynecological diseases; uterus size ≤12 weeks | VH  AH  LH | 50  50  50 | 0  1  0 | 0  0  0 | 0  1  0 |
| Olsson | 1996 | Sweden | Benign gynecological diseases; | LAVH  AH | 71  72 | 1  1 | 0  0 | 1  1 |
| Ottosen | 2000 | Sweden | Fibroma or Myoma; CIN or cervical disease; Dysmenorrhea or Pelvic pain; Uterus size ≤16 weeks | VH  AH  LAVH | 40  40  40 | 1  0  0 | 0  0  0 | 1  0  0 |
| Panda* | 2015 | India | Fibroma or Myoma; Adenomyosis; Hyperplasia; CIN or cervical disease; Endometriosis or endometrial disease; Uterus size ≤16 weeks | VH  AH  LAVH | 49  50  46 | 0  0  0 | 0  0  0 | 1  1  0 |
| Paraiso | 2013 | USA | Fibroma or Myoma; Dysfunctional uterine bleeding; Ovarian cyst; Endometriosis or endometrial disease; Dysmenorrhea or Pelvic pain | LH  RH | 26  26 | 0  0 | 0  0 | 0  0 |
| Park | 2021 | Korea | Fibroma or Myoma; Adenomyosis; Hyperplasia; Dysfunctional uterine bleeding; CIN or cervical disease | SPLH  vNOTES | 13  13 | 0  0 | 0  0 | 0  0 |
| Parveen* | 2013 | Pakistan | Benign gynecological diseases; uterus size ≤14 weeks | VH  AH | 38  38 | 0  0 | 0  0 | 0  1 |
| Perino | 1999 | Italy | Benign gynecological diseases; uterus size ≤16 weeks | LH  AH | 51  51 | 0  0 | 1  0 | 1  0 |
| Persson | 2006 | Sweden | Menometrorrhagia, dysmenorrhoea, dysplasia of the cervix, endometrial hyperplasia without atypia, ﬁbroids or other benign gynaecological dis-eases | LAVH  AH | 63  56 | 1  0 | 0  0 | 1  0 |
| Raju | 1994 | UK | Benign gynecological diseases | LAVH  AH | 40  40 | 0  0 | 0  0 | 0  0 |
| Ribeiro | 2003 | Brazil | Fibroma or Myoma; Adenomyosis | VH  AH  LH | 20  20  20 | 1  0  0 | 0  0  0 | 1  0  0 |
| Richardson | 1995 | UK | Benign gynecological diseases; uterus size ≤16 weeks | LH  VH | 22  23 | 1  1 | 0  0 | 1  1 |
| Roy | 2011 | India | Benign gynecological diseases; Adenomyosis; Hyperplasia; CIN or cervical disease;  uterus weighs < 400 g | RCT, 3arms | VH | 0  0  0 | 0  0  0 | 0  0  0 |
| Roy | 2012 | India | Fibroma or Myoma; Adenomyosis; uteri size=12-24weeks | LAVH  VH | TLH | 0  0 | 0  0 | 0  0 |
| Sarlos | 2012 | Switzerland | Benign gynecological diseases; uterus weighs < 500 g | LH  RH | LAVH | 0  0 | 1  0 | 1  0 |
| Schutz | 2002 | Germany | Benign gynecological diseases , estimated uterine weight of >200 g | LAVH  AH | 28  20 | 0  0 | 0  0 | 0  0 |
| Seracchioli | 2002 | Italy | Benign gynecological diseases; uteri estimated to weigh at least 300g | AH  LH | 61  60 | 1  0 | 0  0 | 1  0 |
| Sesti | 2008a | Italy | Fibroma or Myoma; uteri size≥12weeks and <16weeks | LAVH  VH | 40  40 | 0  0 | 0  0 | 0  0 |
| Sesti | 2008b | Italy | Fibroma or Myoma; uteri size≥12weeks and <16weeks | AH  LAVH  VH | 50  50  50 | 0  0  0 | 0  0  0 | 0  0  0 |
| Sesti | 2014 | Italy | Fibroma or Myoma; uteri size≥12weeks and <16weeks | LH  LAVH | 36  36 | 0  0 | 0  0 | 0  0 |
| Song | 2013 | Korea | Fibroma or Myoma; Adenomyosis; uteri size ≤20week | LH  SPLH | 19  20 | 0  0 | 0  0 | 0  0 |
| Summit* | 1992 | USA | Benign gynecological diseases | LH  VH | 29  27 | 0  0 | 0  0 | 1  0 |
| Summit | 1998 | USA | Benign gynecological diseases | LAVH  AH | 34  31 | 1  1 | 0  0 | 1  1 |
| Tormena | 2017 | Brazil | Benign gynecological diseases | SPLH  LH | 21  19 | 0  0 | 0  0 | 0  0 |
| Tsai | 2003 | China (Taiwan) | Fibroma or Myoma; Adenomyosis; Endometrial hyperplasia; CIN or cervical disease; Endometriosis or endometrial disease; Chronic pelvic pain | LAVH  AH | 100  100 | 0  1 | 0  0 | 0  1 |
| Wakhloo | 2015 | India | Fibroma or Myoma; Dysfunctional uterine bleeding; Ovarian cyst | LH  AH | 50  50 | 2  0 | 2  0 | 4  0 |
| Yuen | 1998 | Hong Kong | Benign gynecological diseases, uterus size ≤16 weeks | AH  LAVH | 24  20 | 0  0 | 0  0 | 0  0 |
| Zhou | 2009 | China | Fibroma or Myoma, uterus size ≤12 weeks | AH  VH  LAVH | 32  34  35 | 0  0  0 | 0  0  0 | 0  0  0 |

**Intervention**: **AH**: abdominal hysterectomy, **LAVH**: laparoscopically assisted vaginal hysterectomy, **LH**: total laparoscopic hysterectomy, **VH**: vaginal hysterectomy, **SPLH**: single-port laparoscopic hysterectomy, **RH**: robotic hysterectomy, and **vNOTES**: vaginal natural orifice transluminal endoscopic surgery.

* Studies in which the outcome was generically reported as urological damage.
