# Supplementary material for: Revisiting Urological Injury Risks in Minimally Invasive Hysterectomy for Benign Indications: A Rare-Events Network Meta-Analysis with Sensitivity Analyses
Source: Int Urogynecol J. 2026 Mar 25;37(9):2791–8. doi: 10.1007/s00192-026-06566-y (PMC13649814; doi:10.1007/s00192-026-06566-y)
Supplement: Supplementary file 4 [file 192_2026_6566_MOESM7_ESM.docx]

| **Surgical approach** | **Injury rate per 1000 procedures** | **Approximate rate (1 in x procedures)** | **95% CI (range, 1 in x)** |
| --- | --- | --- | --- |
| Abdominal hysterectomy (AH) | 7.47 | 1 in 134 | 1 in 227 – 1 in 79 |
| Vaginal hysterectomy (VH) | 10.64 | 1 in 94 | 1 in 153 – 1 in 58 |
| Laparoscopic-assisted vaginal hysterectomy (LAVH) | 10.62 | 1 in 94 | 1 in 152 – 1 in 59 |
| Total laparoscopic hysterectomy (LH) | 18.10 | 1 in 55 | 1 in 95 – 1 in 32 |
| Robotic hysterectomy (RH) | 11.54 | 1 in 87 | 1 in 270 – 1 in 28 |
| vNOTES | 20.83 | 1 in 48 | 1 in 343 – 1 in 7 |
| Single-port laparoscopic hysterectomy (SPLH) | 0.00 | No events reported | – |

ESM7: Crude rate of bladder/ureter injuries according to surgical approach: information for preoperative counselling.
